# Physician assistant for gynecology – exploring awareness and acceptance in Germany

**DOI:** 10.1186/s12913-025-13375-4

**Published:** 2025-09-22

**Authors:** Sophia Andres, Katharina Hancke

**Affiliations:** Department of Obstetrics and Gynecology, University of Ulm, Prittwitzstr. 43, Ulm, 89075 Germany

**Keywords:** Physician assistant (PA), Gynecology, Job description, Areas of application

## Abstract

**Background:**

The Physician Assistant (PA) bachelor’s degree program has been available in Germany for several years, and PAs are progressively being hired and assigned to hospitals. Although the German Medical Association has developed a curriculum, the specific roles and potential duties of PAs in hospitals remain undefined. This study aims to explore the various applications and responsibilities of PAs within the field of gynecology.

**Methods:**

Between December 2023 and February 2024, a cross-sectional, web-based, and anonymized survey was sent to gynecologists via the mailing lists of the Young Forum of the German Society of Gynecology and Obstetrics (DGGG) and through their Instagram account. A total of 120 surveys were collected, of which 97 were completed. The topics of the questionnaire were related to the expected integration of PAs in routine working tasks in the hospital.

**Results:**

This study shows that a significant proportion of respondents considered administrative duties (80%), blood sampling (95%), inserting intravenous catheters (95%), taking medical histories (81%), and conducting ward rounds under medical supervision (65%) to be suitable tasks for Physician Assistants (PAs). In contrast, procedures such as transvaginal sonography (22%), sonographic interventions (15%), and educational tasks (31%) were deemed unsuitable for delegation to PAs.

**Conclusion:**

The introduction of PAs in gynecology may be integrated into everyday clinical practice, but the specific tasks should be clearly defined. Establishing these conditions may have the potential to enhance job satisfaction for doctors.

**Supplementary Information:**

The online version contains supplementary material available at 10.1186/s12913-025-13375-4.

## Background

Despite the global spread of the Physician Assistant role, its integration into German clinical care was introduced in 2017 and remains underrepresented in the scientific literature. The profession of Physician Assistant (PA) was first introduced in the United States in the 1960 s to address the shortage of physicians in primary care [1]. The aim was to support physicians by taking over technical tasks, allowing them to concentrate on their core responsibilities. In Europe, the profession was established in the Netherlands (2001) and the United Kingdom (2004) [1]. In 2017, the German Medical Association (Bundesärztekammer [BÄK]), the Association of Statutory Health Insurance Physicians (Kassenärztliche Vereinigung [KV]), the German Academic Association of Physician Assistants (Deutscher Hochschulverband Physician Assistants e.V. [DHPA]), and the German Society of Physician Assistants (Deutsche Gesellschaft für Physician Assistants e.V. [DGPA]) developed a conceptual framework for the PA profession in Germany [1–3]. Similar to its introduction in the USA, the PA profession was intended to mitigate the impending physician shortage, due to demographic changes and altered working hour models [4].

Increasing documentation and administrative demands have contributed to declining job satisfaction among physicians and heightened the need for delegable tasks. The introduction of PAs allows non-physician staff to assume medical responsibilities, potentially improving physician job satisfaction [3].

The PA degree program is structured as a Bachelor’s program leading to a Bachelor of Science (B.Sc) degree [1]. Since its implementation in 2005, approximately 22 universities now offer this program, according to the DGPA [5]. Graduates primarily work in hospitals, especially in surgical departments [6]. According to the BÄK and KV framework, PAs are entrusted with contributing to diagnostics, treatment planning, and implementation, performing complex examinations, emergency care, communication, process management, and documentation [3]. However, it is important to note that PAs do not undertake the full spectrum of medical activities. Tasks are delegated by physicians, who retain overall responsibility [3]. Delegating physicians must ensure that the PA’s qualifications (e.g., training certification, bachelor’s degree) meet the requirements.

The specialty of gynecology covers all gender-specific health issues, including prevention, diagnosis, conservative and surgical treatment, and follow-up care [7]. This includes subspecialties such as gynecological oncology, endocrinology, reproductive medicine, urogynecology, and plastic-reconstructive surgery, as well as obstetrics, which involves prenatal care, childbirth, and postnatal care [8].

Training in gynecology and obstetrics requires rotations across various departments to meet competency requirements and minimum case numbers, which depend on patient volume, intervention frequency, and the availability of supervising physicians [8]. However, concerns exist that the required competencies may not be achieved within the stipulated 60 months, as compliance with the European Worktime Directive limits participation in routine activities due to reduced working hours and rest requirements [9]. High demands of night and shift work exacerbate these challenges.

Therefore, the introduction of PAs raises concerns regarding trainees` qualification process due to competition for training resources and the achievement of minimum case numbers aggravating this situation.

This study aimed to investigate how gynecological trainees and residents in Germany perceive the role of PAs in gynecology and obstetrics.

## Methods

### Aim, study design, and setting

This study aimed to assess physicians’ expectations of physician assistants (PAs), including their attitude towards the delegation of clinical administrative tasks, as well as any perceived professional conflicts related to overlapping responsibilities or competition for training opportunities. A cross-sectional, web-based, anonymized survey was conducted between December 2023 and February 2024. The study was conducted nationwide in Germany and targeted physicians in training and early-career gynecologists. The survey was disseminated through the email distribution list and Instagram account of the Young Forum of the German Society of Gynecology and Obstetrics (DGGG), as well as via personal networks at university women’s hospitals.

### Participants and materials

The target population included physicians in postgraduate training in gynecology and obstetrics. Access to this group was made possible via the DGGG’s Young Forum, which maintains a distribution list of approximately 1,000 physicians in training. The questionnaire was specifically developed for this study and has not been published previously. It is provided in Supplement 1.

### Questionnaire development and procedures

The questionnaire design was based on the methodological recommendations of The Effective Survey by Günter Lehmann [10]. The development process involved three steps: defining the study objectives, designing the survey instrument, and conducting a pre-test to improve its quality and validity.

The final questionnaire used a structured format and consisted primarily of closed-ended items, including Likert-scale questions, multiple-choice items, and ranking tasks. These were designed to ensure consistency in responses and to facilitate quantitative analysis. The online tool SurveyMonkey (Momentive Inc., USA) was used for questionnaire distribution using a hyperlink.

Before launch, a pre-test was conducted with six physicians at the University Women’s Hospital in Ulm. Participants were asked to evaluate the clarity of the questions, estimated completion time, and the relevance of the content. Based on their feedback, the questionnaire was refined. Given the small sample size of the pre-test group, no statistical analysis was applied to pre-test data.

### Sample size and recruitment

Due to the lack of reliable data on the total number of trainees in gynecology in Germany, the sample size calculation was based on the estimated number of reachable contacts (*N* = 1000). Using the Promidis formula (n ≥ N/[1 + e²*N]) with a margin of error of 10%, the minimum number of responses required for a representative sample was 91 [10].

Recruitment began in early December 2023, with the initial email invitation yielding 27 responses after four weeks. A reminder was subsequently issued, and additional outreach was conducted through personal contacts at university hospitals. By 11 February 2024, a total of 120 responses had been collected. All responses submitted after this date were excluded from analysis.

### Statistical analysis

Data analysis commenced on 11 February 2024. Of the 120 collected questionnaires, 96 were fully completed and included in the final analysis. Participants were allowed to skip individual questions. This study was designed as a descriptive, exploratory, and hypothesis-generating pilot study. Therefore, no hypothesis-driven a priori power or sample size calculations were performed. Statistical analysis was limited to descriptive methods using Microsoft Excel (Microsoft Corp., USA), with results presented as frequencies and percentages. No inferential statistical techniques were applied.

## Results

Approximately one-third of respondents reported that a PA was already employed at their institution (Table 1).

**Table 1:** Demographic data of respondents

	All respondents *N* = 97
In training	54% (*n* = 52)
Resident or specialist	46% (*n* = 45)
Female^a^	87% (*n* = 84)
Male^a^	13% (*n* = 13)
Working in a university hospital	71% (*n* = 69)
Working in a non-university hospital	29% (*n* = 28)
Is there a PA employed in your hospital	
Yes	33% (*n* = 31)
No	68% (*n* = 66)

^a^No answers gender-divers

Most respondents viewed PAs as a valuable addition to the healthcare system. Most respondents were female and worked in university hospitals. (Table 2).

**Table 2:** Perceived usefulness of PAs in physicians’ daily clinical practice

Do you think that the PA in your department is a useful addition to your work as a doctor?			
Respondents with PA in the hospital (***n*** = 31)		Respondents without PA in the hospital (***n*** = 66)	
Yes 94% (*n* = 29)	No 6% (*n* = 2)	Yes 88% (*n* = 58)	No 11% (*n* = 7)^a^

^a^*n* = 1 didn´t know

Respondents were geographically distributed across Germany, with the largest proportion from Southern Germany (52%), followed by Central (20%), Northern (9%), and Eastern Germany (8%).

### Task perception and delegation to PAs

**Fig. 1:**
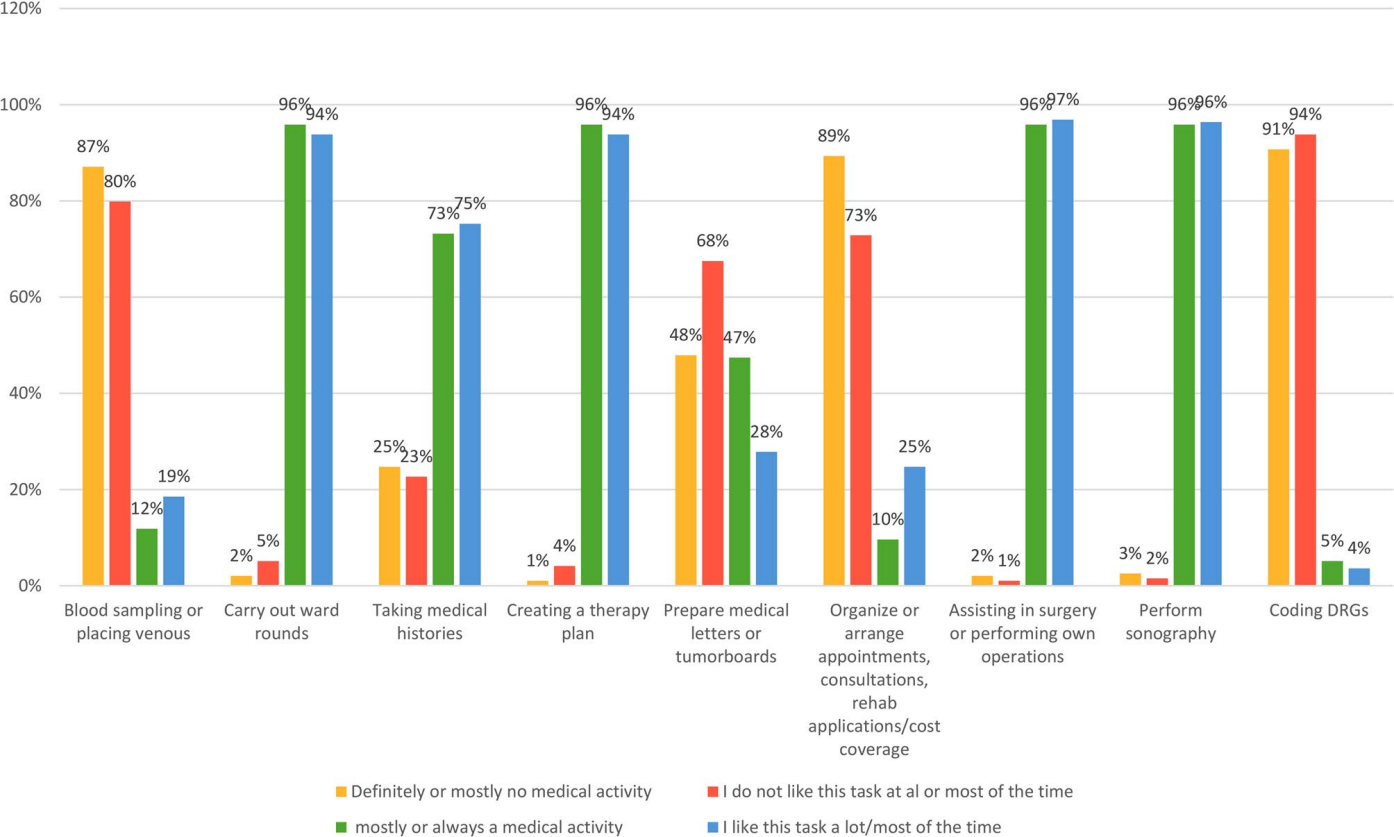
Clinical Tasks Categorized by Perceived Medical Relevance and Personal Preference: There is a consistent association: tasks perceived as medical are more likely to be personally preferred, whereas tasks deemed non-medical tend to be less preferred

Tasks identified as medical correlated closely with those physicians preferred to perform, while tasks physicians preferred to avoid were considered non-medical and deemed appropriate for delegation to PAs (Fig. 1; Table 3).

**Table 3:** Tasks considered to be done by PAs

PAs in our hospital do the following tasks or if no PA is employed, I could imagine PAs being able to take on the following tasks independently after a good induction.							
	Yes, always and partially		rather not and not at all		Not decided		total
	n	%	n	%	n	%	
Blood sampling	92	95%	4	4%	1	1%	97
Placing venous accesses	92	95%	4	4%	1	1%	97
Carry out rounds independently	30	31%	64	66%	2	2%	96
Perform rounds under medical supervision	63	65%	32	33%	1	1%	96
Taking medical histories	79	81%	16	16%	2	2%	97
Prepare medical letters	93	96%	3	3%	1	1%	96
Prepare tumor boards	80	82%	14	14%	3	3%	97
Carry out educational talks	30	31%	63	65%	3	3%	96
Wound care	71	73%	25	26%	1	1%	97
Fill out rehab applications, cost transfers	78	80%	15	15%	4	4%	97
Carry out minor examinations (e.g. vaginal stump check after HE before discharge)	44	45%	47	48%	6	6%	97
Transabdominal sonography (e.g. renal sonography/assessment of ascites)	45	46%	49	51%	3	3%	97
Perform sonographic interventions (e.g. ascites/pleural puncture)	15	15%	77	79%	4	4%	96
Transvaginal sonography	21	22%	69	71%	6	6%	97

The evaluation of clinical tasks revealed varying levels of support for delegation to physician assistants (PAs) across different activity types. In the category of non-medical tasks, most respondents considered activities such as blood sampling and venous access placement to be appropriate for PAs. There was also broad support for PAs participating in ward rounds under medical supervision. However, independent ward rounds were viewed more critically, receiving only limited approval (Table 3).

Concerning administrative and preparatory tasks, responsibilities such as preparing tumor board documentation, drafting medical letters, processing rehabilitation applications and cost coverage documents, taking medical histories, and performing wound care were widely accepted as suitable for PAs.

For minor procedures and diagnostic examinations, opinions were more divided. Approximately equal numbers of respondents supported and opposed allowing PAs to inspect the vaginal stump following a hysterectomy. Fewer than half of the participants supported PAs conducting transabdominal sonography, and the majority were opposed to PAs performing either sonographic interventions (e.g., ascites or pleural punctures) or transvaginal sonography.

**Fig. 2:**
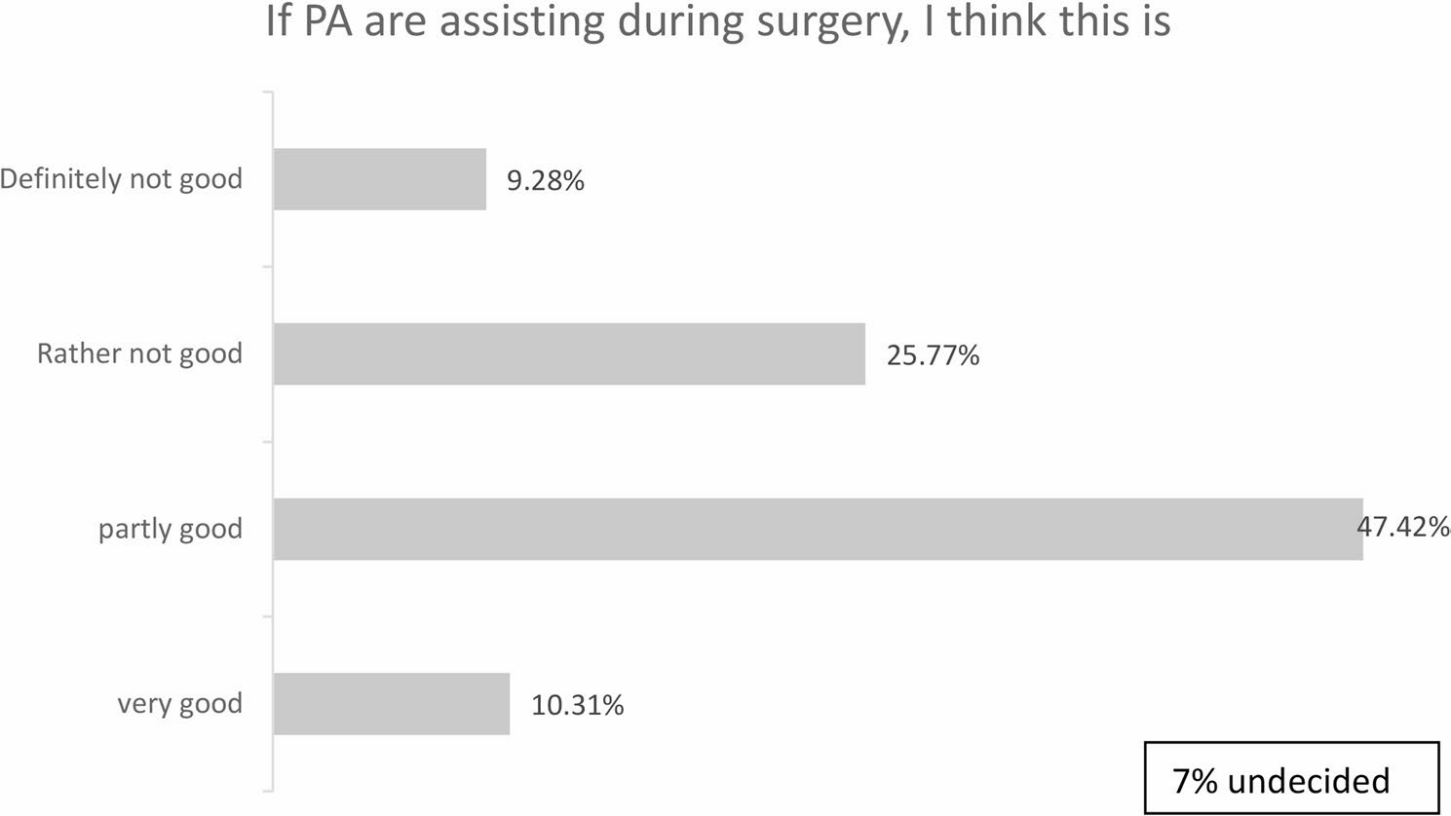
PAs in the operating theatre, *n* = 97

**Fig. 3:**
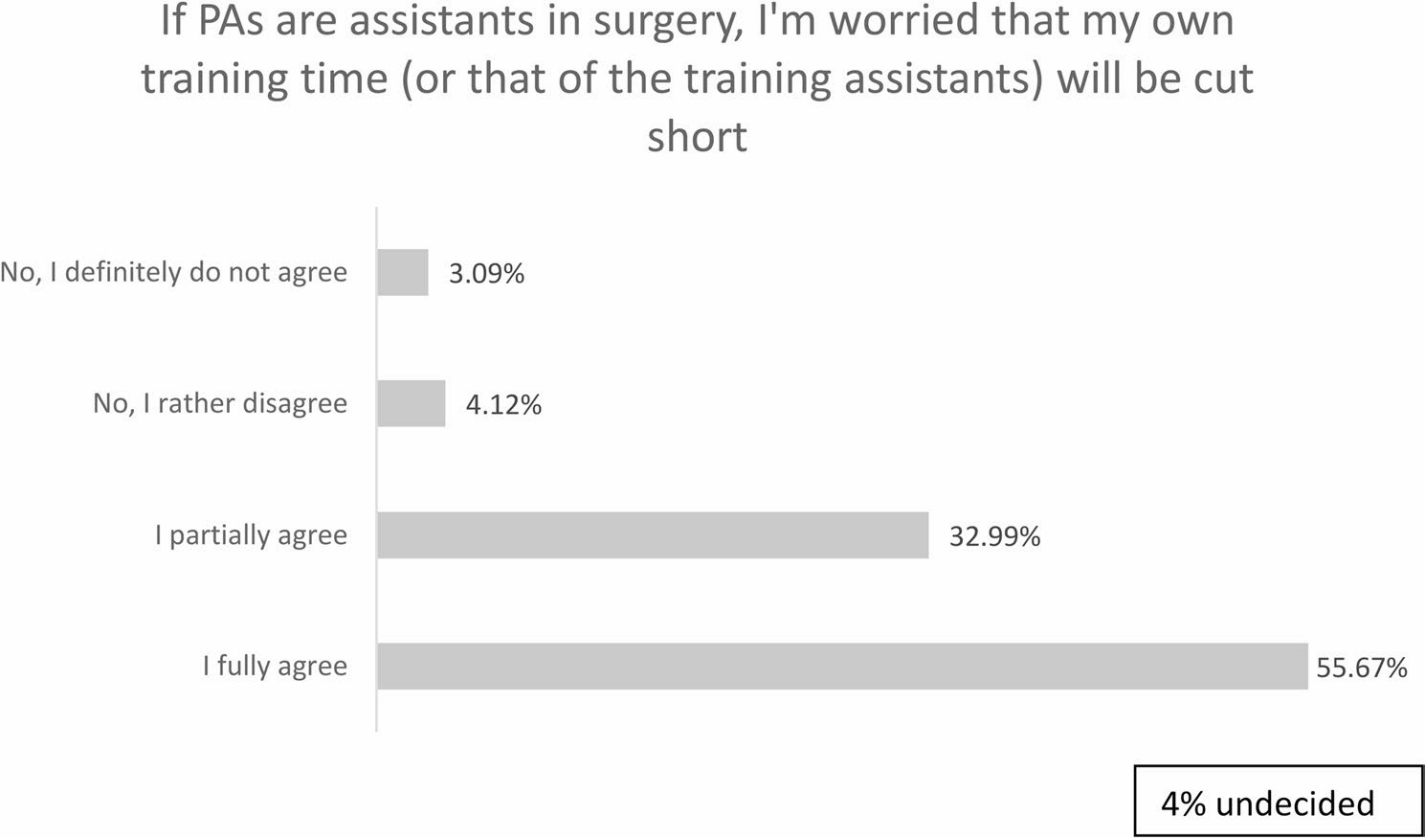
Worries about training time

Regarding surgical assistance, most respondents supported the involvement of PAs in surgical procedures. Nevertheless, many raised concerns that this could adversely affect the training opportunities of medical staff, as reflected in the responses associated with Figs. 2 and 3.

## Discussion

This is the first study addressing the acceptance and awareness of PAs in a gynecological setting in Germany. The results show that PAs are generally well-accepted by medical professionals, with a variety of tasks deemed suitable for delegation to PAs. These findings align with previous research indicating that PAs are perceived as valuable in taking over routine and administrative duties, thereby reducing physician workload [11]. Most respondents identified blood sampling and venous access as appropriate responsibilities for PAs. Additionally, documentation-related tasks—such as preparing tumor board reports, drafting discharge letters, managing rehabilitation applications, processing cost transfers, and taking patient histories—were perceived as well-suited for PAs. These findings align with results from a survey by Schneider et al. [12]. Conversely, PAs themselves expressed a preference for responsibilities such as assisting in surgery and wound care, underscoring a potential mismatch in task preferences. Implications for PA job satisfaction in the context of clarity of roles and collaborations with physicians have been discussed by Treusch et al. [13].

Similarly, our results revealed general support for PAs taking over wound care but limited enthusiasm for their involvement in the operating room (OR). While over half of respondents were open to PAs assisting in surgery, nearly 90% expressed concerns that this could negatively impact their surgical training. This apprehension highlights potential areas of conflict between PAs and physicians. Similar concerns about competition for hands-on training have been reported in the German context, where physicians in training expressed uncertainty about the long-term implications of PA integration [4].

Training in transvaginal and transabdominal sonography emerged as another contentious issue. Slightly fewer than half of respondents considered transabdominal sonography appropriate for delegation to PAs, while the majority opposed PAs performing procedures such as ascites or pleural punctures. Transvaginal sonography was similarly regarded as unsuitable for PAs by most respondents. These views may reflect concerns over training opportunities or a belief that these tasks are inherently medical and should remain under physician oversight.

The delegation of medical tasks and the training of PAs present potential conflicts between professional groups. While German professional law permits the delegation of certain medical responsibilities, the specific scope of these tasks remains undefined, relying heavily on the qualifications of the PA and the trust established between the delegating physician and the PA [11, 12]. This contrasts with the U.S. system, where the introduction of PAs in the 1960 s was accompanied by clear definitions of their roles [14, 15]. Recently, some German universities have introduced Master’s degree programs for PAs [16]. This raises questions about how responsibilities should differ between Bachelor’s and Master’s graduates, potentially intensifying training-related tensions.

Another unresolved issue is the financial classification of PAs in Germany. Unlike in the U.S [15, 17], where PAs are assigned to a standardized salary scale, German PAs are classified according to collective bargaining agreements. At university hospitals, PAs are typically categorized in salary groups E9 or E10, equivalent to positions such as nursing staff in leadership roles (e.g., ward managers) or social workers with a university degree [4, 18].

The question of night shift participation also remains contentious. Since PAs are officially required to work under supervision, they rarely contribute during night shifts. This limitation raises concerns about the equitable distribution of workload, particularly as night shifts are often viewed as onerous and detrimental to quality of life. The prospect of PAs earning comparable salaries to master’s graduates while being exempt from night shifts could potentially undermine the attractiveness of the medical profession.

### Future research

Future studies should aim to collect data from a broader and more representative sample of gynecology departments, including all levels of patient care. In-depth qualitative studies could further explore physicians’ and PAs’ perspectives on role boundaries, trust, and perceived conflicts in daily clinical practice. Since the PA profession was established to reduce physicians’ workload, nationwide studies should be conducted to evaluate this effect from an economic perspective. Furthermore, the work of PAs should be compared across different hospital settings and their efficiency assessed to develop a more standardized training curriculum for this emerging professional role.

### Strengths and limitations

The study’s low response rate is a significant limitation, likely attributable to the heavy workload of physicians and their limited availability to complete surveys. Most respondents were affiliated with university hospitals, suggesting that scientific interest may have motivated their participation more than in non-university settings. While the response rate constrains the generalizability of the findings, this study provides preliminary insights into the integration of PAs in gynecological settings. We did not include questions for the management of obstetric care. This was because PAs in Germany are usually implemented in a gynecological setting. And this, in turn, is because midwifery students have a comparable level of further training. However, this is a strength of the study, as it focuses on work and classification in gynecological settings. Another strength of this study is that this is the first study to reflect the perception and assessment of gynecological physicians of the PA profession.

## Conclusion

This study directly addressed its core aim by capturing physicians’ expectations of PAs, their acceptance of role integration, and their attitudes toward the delegation of specific tasks. The findings reveal a generally positive perception of PAs as supportive team members, particularly for administrative and routine clinical duties. However, concerns regarding training competition and role boundaries—especially in surgical and diagnostic domains—highlight underlying tensions that need to be considered when defining interprofessional roles in gynecology.

## Supplementary Information

Supplementary Material 1.

## Data Availability

The data in this study were collected for the preparation of a master’s thesis and are partially documented in this thesis [19]. If deemed relevant, further datasets used during the current study are available from the corresponding author on reasonable request.

## Abbreviations

BÄK: the German Medical Association (Bundesärztekammer)
B. Sc: Bachelor of Science
DHPA: German Academic Association of Physician Assistants (Deutscher Hochschulverband Physician Assistants e.V.)
DGPA: German Society of Physician Assistants (Deutsche Gesellschaft für Physician Assistants e.V.)
KV: the Association of Statutory Health Insurance Physicians (Kassenärztliche Vereinigung)
OR: Operating room
PA: Physician Assistant

## References

[1] Wessels M, Geuen M. Physician Assistant. Ein Gesundheitsberuf etabliert sich im deutschen Gesundheitswesen. Vol. Band 3. Berlin: LIT Verlag Dr. W. Hopf; 2021. 378 p. ISBN: 978-3-643-14756-1.

[2] 120. Deutscher Ärztetag. Beschlussprotokoll. 120. Deutscher Ärztetag. Beschlussprotokoll. 2017.

[3] Bundesärztekammer K. Physician Assistant - ein Neuer Beruf Im Deutschen gesundheitswesen. Bundesärztekammer und Kassenärztliche Bundesvereinigung; 2017.

[4] Erlenberg RM, Günther HJ, Heistermann HP, Sesselmann SM. Assistenzärzte und physician assistants: eine alternative Zur behebung des ärztemangels?? ZFPG. Jg / Nr. 2021;2:24–31. 10.17193/HNU.ZFPG.07.02.2021-05.

[5] DGPA. DGPA. [cited 2022 Aug 14]. Berufsbild Pa | DGPA Deutsche Gesellschaft für Physician Assistants e.V. Available from: https://www.pa-deutschland.de/berufsbild-pa

[6] Heistermann P, Günther HJ, Heilmann C, Meyer-Treschan T, Sesselmann S, Schneke A, et al. A cross-sectional survey of German PA employment and workforce entry. JAAPA. 2022;35(12):45–9. 10.1097/01.JAA.0000892728.78698.75. 36350301 10.1097/01.JAA.0000892728.78698.75

[7] Deutsche Gesellschaft für Gynäkologie und Geburtshilfe e. V. Kontakt [Internet]. Werden (DE): GYN-WERDEN. Available from: https://www.gyn-werden.de/kontakt. Cited 2025 Sep 13.

[8] Weiterbildungsordnung (WBO). Baden-Württemberg. Weiterbildungsordnung (WBO) der Landesärztekammer Baden-Württemberg vom 18. Mai 2020. 2020;448.

[9] Hancke K, Igl W, Toth B, Bühren A, Ditsch N, Kreienberg R. Work-life balance of German gynecologists: a web-based survey on satisfaction with work and private life. Arch Gynecol Obstet. 2014;289(1):123–9. 10.1007/s00404-013-2949-y. 23860692 10.1007/s00404-013-2949-y

[10] Lehmann, die effektive Befragung. 3.überarbeitete Ausgabe. 14.11.2022. Tübingen

[11] Halter M, Drennan V, Chattopadhyay K, Carneiro W, Yiallouros J, de Lusignan S, et al. The contribution of physician assistants in primary care: a systematic review. BMC Health Serv Res. 2013;13:223. 10.1186/1472-6963-13-223. 23773235 10.1186/1472-6963-13-223 PMC3698179

[12] Schneider S, Stengel D, Seifert J, Ekkernkamp A, Ludwig J. [Perceived utility of the inclusion of physician assistants in the surgical process quality and continuing education in germany: results of an interprofessional online survey]. Unfallchirurgie (Heidelb). 2024;127(6):457–68. 10.1007/s00113-024-01431-3. 38668769 10.1007/s00113-024-01431-3

[13] Treusch Y, Möckel L, Kohlstedt K. Working conditions, authorizations, mental health, and job satisfaction of physician assistants in Germany. Front Public Health. 2023;11:1082463. 10.3389/fpubh.2023.1082463. 36908456 10.3389/fpubh.2023.1082463 PMC9998044

[14] AAPA History. 2023. https://www.aapa.org/about/history/

[15] Cawley JF, Cawthon E, Hooker RS. Origins of the physician assistant movement in the united States. JAAPA. 2012;25(12):36–40. 10.1097/01720610-201212000-00008. 23600002 10.1097/01720610-201212000-00008

[16] DGPA e.V. [Internet]. [Cited 2025 Jul 17]. Studium Physician Assistance. Available from: https://www.pa-deutschland.de/studium

[17] Research [Internet]. AAPA. [Cited 2025 Jul 17]. Available from: https://www.aapa.org/research/

[18] TdL. Tarifverträge [Internet]. [Cited 2025 Jul 12]. Available from: https://www.tdl-online.de/tarifvertraege

[19] Hancke K. Physician Assistant – Berufsbild, aufgaben und möglichkeiten in der Frauenheilkunde in Deutschland und Im internationalen vergleich (unveröffentlichte Masterarbeit). HNU (Hochschule Neu Ulm); 2024.

