# Supplementary material for: Physician assistant for gynecology – exploring awareness and acceptance in Germany
Source: BMC Health Serv Res. 2025 Sep 22;25:1205. doi: 10.1186/s12913-025-13375-4 (PMC12451998; doi:10.1186/s12913-025-13375-4)
Supplement: Supplementary file 1 — Supplementary Material 1. [file 12913_2025_13375_MOESM1_ESM.docx]

Survey “Physician Assistants in Germany”

English Translation of German Original

Thank you very much for taking part in this survey – it is about your assessment of the new profession “Physician Assistant” (PA) and its possible deployment in gynaecology. The degree programme has existed in Germany since 2005, but at the present time there are only a few PAs employed in women’s hospitals or gynaecology departments. Physician Assistants are assigned to the medical profession and, after appropriate induction, may be delegated and carry out medical tasks such as blood draws, ward rounds, medical histories, preparation of diagnostics or treatment plans/tumour boards, discharge examinations, ultrasounds or assistance in surgery. In principle the medical teams/superiors decide which medical tasks are to be delegated.

With this questionnaire I would like to find out, firstly, whether you can imagine how PAs could be deployed in gynaecology and, secondly, whether you could think of conflicts between the professional groups. Completing the questionnaire will take less than 10 minutes of your time and is of course anonymous.

If you have any questions, please feel free to contact me by e‑mail: XXXX

1. Are you already familiar with the possible areas of deployment of Physician Assistants (PAs)?

☐ Yes, we have a PA in our department

☐ Yes, I know the profession, but at present no PA is working in our department

☐ No, I have no knowledge of the possible areas of deployment of PAs

2. Do you think that a PA in your department is a useful complement to your medical work?

☐ Yes, very much so

☐ Yes, partly

☐ Rather not

☐ No, not really

☐ Other (please specify): __________

3. Which of the following tasks does the PA in your department currently perform?

|  | Yes, always | Yes, sometimes | Rather not | Not at all | I am indifferent |
| --- | --- | --- | --- | --- | --- |
| Blood draws |  |  |  |  |  |
| Inserting venous access lines |  |  |  |  |  |
| Conducting ward rounds independently |  |  |  |  |  |
| Conducting ward rounds under medical supervision |  |  |  |  |  |
| Taking medical histories |  |  |  |  |  |
| Preparing discharge / medical letters |  |  |  |  |  |
| Preparing tumour boards |  |  |  |  |  |
| Conducting informed‑consent discussions |  |  |  |  |  |
| Wound care |  |  |  |  |  |
| Completing rehabilitation applications / cost‑coverage forms |  |  |  |  |  |
| Performing minor examinations (e.g. check of vaginal stump after hysterectomy before discharge) |  |  |  |  |  |
| Transabdominal ultrasound (e.g. nephrosonography / assessment of ascites) |  |  |  |  |  |
| Performing sonographically guided interventions (e.g. ascites / pleural puncture) |  |  |  |  |  |
| Transvaginal ultrasound |  |  |  |  |  |

4. The PA in our department is firmly integrated into the medical team.

☐ Yes, very much so

☐ Yes, partly

☐ Rather not

☐ Not at all

☐ I prefer not to answer

5. Could you imagine that PAs would be a good complement to medical tasks?

☐ Yes, definitely

☐ Yes, partly

☐ Rather not

☐ No, definitely not

☐ I cannot answer that

6. Which of the following tasks could PAs, in your opinion, take over independently after thorough induction?

|  | Yes, always | Yes, sometimes | Rather not | Not at all | I am indifferent |
| --- | --- | --- | --- | --- | --- |
| Blood draws |  |  |  |  |  |
| Inserting venous access lines |  |  |  |  |  |
| Conducting ward rounds independently |  |  |  |  |  |
| Conducting ward rounds under medical supervision |  |  |  |  |  |
| Taking medical histories |  |  |  |  |  |
| Preparing discharge / medical letters |  |  |  |  |  |
| Preparing tumour boards |  |  |  |  |  |
| Conducting informed‑consent discussions |  |  |  |  |  |
| Wound care |  |  |  |  |  |
| Completing rehabilitation applications / cost‑coverage forms |  |  |  |  |  |
| Performing minor examinations (e.g. check of vaginal stump after hysterectomy before discharge) |  |  |  |  |  |
| Transabdominal ultrasound (e.g. nephrosonography / assessment of ascites) |  |  |  |  |  |
| Performing sonographically guided interventions (e.g. ascites / pleural puncture) |  |  |  |  |  |
| Transvaginal ultrasound |  |  |  |  |  |

7. Which of the following tasks, in your opinion, should rather not be delegated to PAs, even after thorough induction?

(Please tick all that apply)

☐ Taking medical histories

☐ Blood draws

☐ Inserting venous access lines

☐ Creating therapy plans

☐ Transabdominal ultrasounds

☐ Transvaginal ultrasounds

☐ Other (please specify): __________

8. If PAs are employed in the operating theatre, I think that is …

☐ Very good

☐ Partly good

☐ Rather not good

☐ By no means good

☐ I am undecided

9. If PAs are employed in the operating theatre, I am concerned that my own operating‑room training time (or that of other residents) will be reduced.

☐ I totally agree

☐ I partly agree

☐ No, I rather disagree

☐ No, I totally disagree

☐ I am undecided

10. How do you rate the deployment of PAs in on‑call or shift duties?

☐ I would definitely find their participation useful

☐ I would partly find their participation useful

☐ I would rather not find their participation useful

☐ I would find their participation by no means useful

☐ I am undecided

11. When you think of your daily work routine, which of the following do you NOT consider to be medical activities?

|  | Definitely not | Mostly not | Mostly medical | Always medical | Don’t know |
| --- | --- | --- | --- | --- | --- |
| Blood draws |  |  |  |  |  |
| Inserting venous access lines |  |  |  |  |  |
| Conducting ward rounds independently |  |  |  |  |  |
| Taking medical histories |  |  |  |  |  |
| Creating therapy plans |  |  |  |  |  |
| Preparing discharge / medical letters |  |  |  |  |  |
| Preparing tumour boards |  |  |  |  |  |
| Organising consultations and requesting findings |  |  |  |  |  |
| Checking study inclusions |  |  |  |  |  |
| Wound care |  |  |  |  |  |
| Scheduling appointments for patients |  |  |  |  |  |
| Assisting in surgery or performing own operations |  |  |  |  |  |
| Performing transabdominal ultrasound |  |  |  |  |  |

12. Which of the following professional activities do or don`t you like to perform?

|  | Like to perform | Mostly like to perform | Mostly don’t like to perfrom | Don`t like to perform | Don’t know |
| --- | --- | --- | --- | --- | --- |
| Blood draws |  |  |  |  |  |
| Inserting venous access lines |  |  |  |  |  |
| Conducting ward rounds independently |  |  |  |  |  |
| Taking medical histories |  |  |  |  |  |
| Creating therapy plans |  |  |  |  |  |
| Preparing discharge / medical letters |  |  |  |  |  |
| Preparing tumour boards |  |  |  |  |  |
| Organising consultations and requesting findings |  |  |  |  |  |
| Checking study inclusions |  |  |  |  |  |
| Wound care |  |  |  |  |  |
| Scheduling appointments for patients |  |  |  |  |  |
| Assisting in surgery or performing own operations |  |  |  |  |  |
| Performing transabdominal ultrasound |  |  |  |  |  |

13. What percentage of your daily work do you estimate is non‑medical activity?


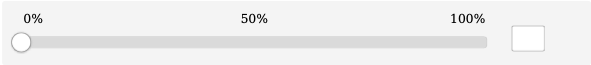


(slider or mark)

14. What percentage of your daily work do you spend on patient‑related tasks (e.g. ward rounds, blood draws, surgical assistance, examinations, etc.)?


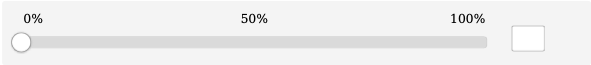


(slider or mark)

15. How many overtime hours per week do you estimate you work due to non‑medical tasks?

☐ None

☐ 1–2 h/week

☐ 3–5 h/week

☐ More than 5 h/week

16. What percentage of your daily working time do you spend on non‑patient‑related tasks (e.g. coding diagnoses, preparing letters/tumour boards, documentation, etc.)?


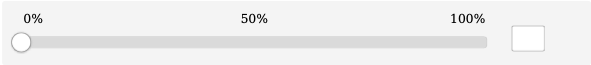


(slider or mark)

17. Thank you for taking the time to answer the questions. Now just a few questions about your personal/demographic data.

Your gender:

☐ Female

☐ Male

☐ Diverse

18. Year of birth: __________

19. In which year of training are you currently?

☐ 1st–3rd year of speciality training

☐ 4th–5th year of speciality training

☐ More than 5th year of speciality training, exam not yet taken

☐ Specialist (board‑certified)

☐ Senior physician in a hospital

☐ Chief physician

☐ Practice owner

20. In which federal state do you currently work?

☐ Baden‑Württemberg

☐ Bavaria

☐ Berlin

☐ Brandenburg

☐ Bremen

☐ Hamburg

☐ Hesse

☐ Mecklenburg‑Western Pomerania

☐ Lower Saxony

☐ North Rhine‑Westphalia

☐ Rhineland‑Palatinate

☐ Saarland

☐ Saxony

☐ Saxony‑Anhalt

☐ Schleswig‑Holstein

☐ Thuringia

21. In which type of institution do you work? If you are currently on parental leave or maternity leave, please use your last workplace.

☐ University hospital

☐ Teaching hospital

☐ Non‑teaching hospital

☐ Private practice

☐ Other

22. Thank you for your time!

Is there anything else you would like to tell me? __________
